# Burden of diseases due to high systolic blood pressure in the Middle East and North Africa region from 1990 to 2019

**DOI:** 10.1038/s41598-024-64563-x

**Published:** 2024-06-13

**Authors:** Saeid Safiri, Seyed Ehsan Mousavi, Kimia Motlagh Asghari, Seyed Aria Nejadghaderi, Reza Aletaha, Mark J. M. Sullman, Kuljit Singh, Ali-Asghar Kolahi, Mohammad Reza Beyranvand

**Affiliations:** 1 Social Determinants of Health Research Center, Department of Community Medicine, Faculty of Medicine, Tabriz University of Medical Sciences, Tabriz, Iran; 2 Clinical Research Development Unit of Tabriz Valiasr Hospital, Tabriz University of Medical Sciences, Tabriz, Iran; 3 Neurosciences Research Center, Aging Research Institute, Tabriz University of Medical Sciences, Tabriz, Iran; 4 Research Center for Integrative Medicine in Aging, Aging Research Institute, Tabriz University of Medical Sciences, Tabriz, Iran; 5 HIV/STI Surveillance Research Center, and WHO Collaborating Center for HIV Surveillance, Institute for Futures Studies in Health, Kerman University of Medical Sciences, Kerman, Iran; 6 Systematic Review and Meta-Analysis Expert Group (SRMEG), Universal Scientific Education and Research Network (USERN), Tehran, Iran; 7 Department of Life and Health Sciences, University of Nicosia, Nicosia, Cyprus; 8 Department of Social Sciences, University of Nicosia, Nicosia, Cyprus; 9 Department of Cardiology, Gold Coast University Hospital, Gold Coast, Queensland Australia; 10 Social Determinants of Health Research Center, Shahid Beheshti University of Medical Sciences, Tehran, Iran

**Keywords:** High systolic blood pressure, Middle East and North Africa, Disability-adjusted life-years, Epidemiology, Global burden of disease, Public health, Epidemiology

## Abstract

High systolic blood pressure (HSBP) is associated with several metabolic and non-metabolic disorders. This research aimed to document the deaths and disability-adjusted life-years (DALYs) attributable to HSBP in the Middle East and North Africa (MENA) region between 1990 and 2019, by age, sex, underlying cause and socio-demographic index (SDI). We used the methodological framework and data drawn from the Global Burden of Disease study 2019 to identify the burden of diseases attributable to HSBP, from 1990 to 2019, in the MENA region. The estimates reported were presented as counts, population-attributable fractions, and age-standardised rates (per 100,000), along with 95% uncertainty intervals. In 2019, 803.6 thousand (687.1 to 923.8) deaths were attributed to HSBP in MENA, which accounted for 25.9% (22.9–28.6%) of all deaths. The number of regional DALYs caused by HSBP in 2019 was 19.0 million (16.3–21.9 million), which accounted for 11.6% (10.1–13.3%) of all DALYs, and was 23.4% (15.9–31.5%) lower than in 1990. The highest age-standardised DALY rate for 2019 was observed in Afghanistan, with the lowest in Kuwait. Additionally, the DALY rate in MENA rose with age for both sexs. Furthermore, a negative linear relationship was found between SDI and the age-standardised DALY rates. The region has a substantial HSBP-related burden. Policymakers and healthcare professionals should prioritize interventions that effectively promote the early detection of HSBP, access to quality healthcare, and lifestyle modifications to mitigate the HSBP burden in the MENA countries.

## Introduction

High systolic blood pressure (HSBP) is a substantial public health issue. In 2019, HSBP was responsible for 10.8 million deaths globally^1^. Hypertension has a number of risk factors, including high salt consumption, inadequate potassium intake, obesity, drinking alcohol, insufficient physical activity, and a poor diet^2^. Moreover, HSBP is associated with several diseases, which include cardiovascular diseases, diabetes, and kidney diseases^3,4^. Economically, hypertension imposes a noteworthy cost burden, with an average cost estimated at $22/month in low- and middle-income nations^5^.

In 2019, the global age-standardised mortality rate caused by HSBP was 138.9 (95% uncertainty interval (UI): 121.3–155.7) and the years lived with disability (YLDs) rate was 258.5 (95% UI: 185.9–331.9) per 100,000 population^3^. In contrast to a 25.2% decrease in the age-standardised disability-adjusted life years (DALYs) rate in the Middle East and North Africa (MENA) region, over the 1990–2019 period^6^, globally the number of DALYs rose by 52.4% over the same period^7^. In MENA, the HSBP-attributable burden was largest among those aged 75–79 years old in 2019 and the HSBP burden was negatively correlated with the sociodemographic index (SDI)^6^.

Prior investigations have examined the burden of cardiovascular diseases and their associated risk factors, spanning the period from 1990 to 2019, at the global, regional, and national levels^1^. Moreover, another study delved into the global statistics concerning the deaths, YLDs, and DALYs associated with HSBP from 1990 to 2019^3^. A similar study reported the HSBP-attributable burden among older adults across the world over the period 1990–2019^7^. In MENA, the burden of metabolic risk factors, which includes HSBP, was also reported between 1990 and 2019^6^, but the study did not focus on HSBP and its attributable diseases^6^. Similar studies have also been conducted at the national level^8,9^. Although previous studies have evaluated the burden of different types of cardiovascular diseases or relevant risk factors at global, regional, and national levels, to the best of our knowledge, no study reported the HSBP-attributable burden globally or specifically in the MENA region in all age groups. In this article, we employ data from the Global Burden of Disease (GBD) study 2019 to present statistics on the deaths and DALYs linked to health conditions and injuries related to HSBP. The study focuses on the countries that comprise the MENA region, during the period 1990–2019, and the data is reported by age, sex, underlying cause, as well as the relationship with SDI.

## Methods

### Overview

The GBD project is an extensive epidemiological initiative that was establish to monitor and evaluate the epidemiological burden of injuries and diseases across the globe. GBD 2019 incorporates information on nearly 370 diseases/injuries and over 80 risk factors. This extensive dataset offers a thorough and comprehensive perspective on the state of global health^10^. The present study focuses on the MENA region and presents the burden attributed to HSBP over the period 1990–2019. There are 21 countries in MENA, which are: Afghanistan, Algeria, Bahrain, Egypt, Iran, Iraq, Jordan, Kuwait, Lebanon, Libya, Morocco, Oman, Palestine, Qatar, Saudi Arabia, Sudan, the Syrian Arab Republic, Tunisia, Turkey, the United Arab Emirates and Yemen. The HSBP attributable burden has been generated using previously reported methodologies^10–12^. For additional information on the estimates, please refer to these websites: https://vizhub.healthdata.org/gbd-compare/ and http://ghdx.healthdata.org/gbd-results-tool.

### Case Definitions and data sources

Continuous systolic blood pressure measurements (in mmHg) were used as the basis for evaluating hypertension^11^. The mean systolic blood pressure was derived from two main sources: (1) a systematic literature review that was conducted during the GBD 2015 study, and (2) data obtained from household surveys and related reports^11^. The GBD 2019 study utilised a vast collection of information and in the systematic literature review, only population-based studies that measured SBP using a sphygmomanometer were included.

Research that included non-representative populations, lacked sufficient methodological detail, or reported implausibly high or low SBP levels, based on expert judgement and data from similar countries, were excluded^11^. Whenever available, individual level blood pressure data were obtained using microdata derived from surveys. The data points were combined for different demographic groups to calculate average estimates within the GBD 5-year age-sex categories^11^. Where microdata was not available, data from surveys and literature sources were utilized, along with their associated measures of uncertainty (i.e., standard error, uncertainty interval, standard deviation, and sample size). However, these sources often focused solely on the prevalence of hypertension within the studied population, omitting details about the level of hypertension^11^. For the modeling of SBP, most of these sources were not utilized, except for the data acquired from the Behavioral Risk Factors Surveillance System (BRFSS), a self-reported survey that was undertaken via the telephone in the United States of America. The reason for this exception is that the BRFSS survey is structured similarly to the National Health and Nutrition Examination Survey (NHANES), which includes a sample that is representative of the population^11^.

### Modeling strategy

The Institute for Health Metrics and Evaluation (IHME) employed spatiotemporal Gaussian process regression (ST-GPR) to predict the average systolic blood pressure for each country and subnational location, sex, and 5-year age categories (beginning at 25 years old), covering the years 1980 to 2019. The selection of covariates was conducted in two steps. Firstly, a set of variables with causal links to SBP was compiled. These variables were identified based on significant associations being found in reputable prospective cohort studies that were published in the scientific literature. Secondly, the predictive validity of all combinations of covariates within the linear model was assessed using the previously selected covariates as a foundation^11^. This process was conducted separately for each sex. The standard deviation of systolic blood pressure was then modelled for the different country and subnational populations, the researchers considered factors such as sex and 5-year age groups, beginning at 25 years old. The estimations were derived from the standard deviations of the person-level data, which was supplemented with information from tabulated data sources^11^. Moreover, a blood pressure adjustment was applied to prevent overestimation caused by measurement error, in addition to diurnal, seasonal, or biological variations^11^.

### Relative risk

IHME estimated the Theoretical Minimum-Risk Exposure Level (TMREL) for SBP, which represents the level of exposure that reduces the possibility of experiencing any HSBP-related diseases to as low as possible. According to a synthesis of prospective cohort studies, the estimated TMREL of SBP falls within the range of 110 to 115 mmHg^11^. These cohort studies showed there was an increased risk of mortality at SBP levels outside this range^13,14^. The relative risk estimates for blood pressure outcomes have remained unchanged since GBD 2016. The results of the Renal Risk Collaboration meta-analysis, which included 2.7 million participants, were used to ascertain the relative risks related to chronic kidney disease^11^. The CALIBRE study, a cohort study that links health records in the United Kingdom, was used to model the relative risk (RR) for cardiovascular outcomes^14,15^. The estimates of relative risk for other outcomes were obtained from: the Asia Pacific Cohort Studies Collaboration (APCSC) and the Prospective Studies Collaboration (PSC)^16^. To analyze and aggregate effect sizes from the studies, DisMod-MR 2.1 was utilised to construct dose–response curves for each outcome related to HSBP^11^. This tool enabled the integration of random effects between studies and the inclusion of data covering diverse age categories. The IHME applied the relative risks consistently across every country, and meta-regression was used to combine data from the three major sources^11^.

### Diseases associated with HSBP

The population-attributable fraction (PAF) was utilised to assess the impact of HSBP on disease and injury prevalence by nation, age category, sex, and year. To estimate the deaths and disability-adjusted life years (DALYs) due to HSBP, the PAFs were multiplied with the estimated death counts or DALYs in each country, specific disease or injury, age category, sex, and year^11^.

The mortality numbers were modelled using the Cause of Death Ensemble model (CODEm) within the Global Burden of Disease 2019 framework^10^. The CODEm algorithm employed multiple separate models to determine the most parsimonious fit, based on all available data and covariates. In addition, predictive validity was assessed for each model and combination of models. The model with the highest predictive validity, particularly in out-of-sample circumstances, was selected.

The estimation of DALYs utilised a three-stage process. Firstly, the years lived with disability (YLDs) were estimated by multiplying the prevalence of each disease or injury severity category by their corresponding disability weights. Next, the calculation of years of life lost (YLLs) for each injury or disease was undertaken. The number of deaths in each age category were multiplied by the estimated number of years of life left, as obtained from the GBD standard life table, in each specific age category. Following this, the YLLs and YLDs were added together to estimate the total DALYs for each disease or injury^11^. The detailed methodology employed to estimate the overall number of deaths and DALYs has previously been published^10,11^. The estimates reported included counts, PAFs, and age-standardised rates (per 100,000), as well as 95% uncertainty intervals (UIs). The 95% UIs were produced by undertaking one thousand draws at each step in the modelling process and consisted of the 25th and 975th values of the numerically ordered iterations^11^.

In order to explore the association between the age-standardised DALY rate of HSBP-related diseases and the Socio-demographic Index (SDI) for all MENA countries, smoothing splines models were used^17^. As an indicator of socio-economic development, SDI ranges from the highest development level^1^ to the lowest development level (0). SDI is constructed by combining the per capita income, educational attainment (≥ 15 years old) and overall fertility rate (< 25 years old). All figures and analyses were carried out with R software (version 3.5.2).

### Ethics approval

This study was approved by the Shahid Beheshti University of Medical Sciences, Tehran, Iran (IR.SBMU.RETECH.REC.1402.044).

## Results

### The Middle East and North Africa region

In 2019, a total of 803.6 thousand deaths (95% UI: 687.1 to 923.8) were due to HSBP, which was 25.9% (22.9 to 28.6) of all deaths in MENA (Table 1). In addition, there were 424.7 thousand deaths (362.1 to 491.1) among males and 378.9 deaths among females in 2019 (Table S1). That same year, HSBP resulted in 19.0 million DALYs (16.3 to 21.9), which accounted for 11.6% (10.1 to 13.3) of the total DALYs in the region, with 10.6 million (9.0 to 12.3) DALYs being among males and 8.4 million (7.2 to 9.7) DALYs in women (Table S2). In 2019, the age-standardised mortality rate attributed to HSBP was estimated to be 219.4 (185.6 to 252.5) per 100,000, which is 23.4% (15.9 to 31.5) lower than the 1990 level of 286.5 [246.7 to 323.4] (Table S3). In addition, the age-standardised DALY rate per 100,000 also declined from 5887.9 (5221.2 to 6582.3) to 4401.9 (3785.9 to 5042.8) between 1990 and 2019, indicating a relative decrease of 25.2% (16.8 to 33.9) (Table S4).

**Table 1:** The burden of diseases attributable to high systolic blood pressure in the Middle East and North Africa region in 2019 and the percentage change in the age-standardised rates during the period 1990–2019 (generated from data available from http://ghdx.healthdata.org/gbd-results-tool).

	Deaths (95% UI)				DALY (95% UI)			
	Counts (2019)	PAF (2019)	ASRs (2019)	% change in ASRs 1990–2019	Counts (2019)	PAF (2019)	ASRs (2019)	% change in ASRs 1990–2019
North Africa and Middle East	803,631 (687,099, 923,807)	25.9 (22.9, 28.6)	219.4 (185.6, 252.5)	− 23.4 (− 31.5, − 15.9)	19,028,033 (16,327,380, 21,927,273)	11.6 (10.1, 13.3)	4401.9 (3785.9, 5042.8)	− 25.2 (− 33.9, − 16.8)
Afghanistan	36,017 (27,027, 45,488)	14.3 (11.8, 16.7)	341.8 (262.2, 421.6)	− 15.6 (− 35, 5.1)	1,019,766 (760,082, 1,334,770)	6 (4.6, 7.4)	7400.8 (5554.9, 9271.6)	− 19.4 (− 39.4, 4.5)
Algeria	58,602 (46,555, 71,314)	29.1 (24.7, 33.3)	235.5 (185.6, 287)	− 40.1 (− 51.8, − 26)	1,222,949 (966,991, 1,502,570)	12.1 (10, 14.4)	3927.6 (3132.5, 4790)	− 43.6 (− 55.7, − 28.9)
Bahrain	825 (652, 1031)	19.3 (16.8, 22.4)	147.8 (115.6, 184.6)	− 55.5 (− 64.2, − 43.5)	24,262 (19,467, 29,874)	8.5 (6.9, 10.3)	2622.3 (2110.9, 3205)	− 59.2 (− 67.1, − 47.7)
Egypt	164,710 (123,672, 209,933)	29.3 (24.4, 33.7)	314.9 (238.4, 403.5)	1.7 (− 22, 28.4)	4,226,663 (3,191,278, 5,446,888)	16 (13.1, 18.9)	6576.1 (4975.9, 8308.1)	4.1 (− 21.1, 32.3)
Iran (Islamic Republic of)	99,939 (86,758, 112,458)	25.6 (22.1, 28.8)	157.8 (135.3, 179.1)	− 35.6 (− 42.3, − 31.3)	2,127,266 (1,913,281, 2,347,736)	10.8 (9.1, 12.4)	2973.4 (2652.2, 3280.2)	− 38.1 (− 43.8, − 33.8)
Iraq	55,716 (44,030, 67,478)	31 (27.8, 33.9)	296.6 (238.4, 350)	− 10.7 (− 29, 8.8)	1,381,459 (1,078,019, 1,706,965)	13.4 (11.2, 15.6)	6025.4 (4796.3, 7308.9)	− 15.5 (− 33.8, 5.2)
Jordan	8168 (6704, 9751)	25.3 (22.3, 28)	168.5 (137.8, 199.5)	− 39 (− 50.5, − 26.5)	201,159 (168,057, 239,001)	9.4 (7.9, 11)	3228.8 (2707.6, 3804.5)	− 40.4 (− 51.7, − 27.1)
Kuwait	2534 (2062, 3083)	25.3 (21.8, 28.4)	115.3 (92.9, 138.7)	− 36.9 (− 46.9, − 25.7)	69,176 (56,649, 83,667)	9.2 (7.5, 11.1)	2379.7 (1967.1, 2861.6)	− 36.5 (− 46.1, − 25.1)
Lebanon	9757 (7074, 11,640)	28.8 (21.2, 33.8)	191.3 (138.6, 228.4)	− 26.3 (− 45.9, − 12)	203,776 (153,914, 240,750)	15.1 (11.3, 17.7)	3904.4 (2956.7, 4605.3)	− 26.3 (− 44.2, − 9.6)
Libya	8654 (6726, 10,717)	27.3 (23.5, 31.3)	188.7 (146.1, 232.3)	− 5.9 (− 26.1, 20.9)	217,945 (172,545, 273,901)	12.8 (10.6, 15.3)	4062 (3223.6, 5040.7)	− 3.6 (− 24.8, 23.7)
Morocco	79,904 (61,938, 93,834)	35 (30.6, 39.4)	307.7 (240, 359.3)	− 9.3 (− 25.7, 5)	1,803,693 (1,394,123, 2,176,429)	17.9 (14.7, 21)	5955.2 (4659.1, 7044.1)	− 14.5 (− 30.7, 1.5)
Oman	2996 (2540, 3494)	24.2 (21, 27.3)	274.5 (225.1, 326.8)	− 4.5 (− 24.1, 23.6)	80,551 (68,395, 94,411)	9.4 (7.8, 11.3)	4921.4 (4161.2, 5715.3)	− 16.8 (− 34.3, 10.2)
Palestine	3816 (3182, 4529)	23 (19.9, 26)	211.6 (174.5, 252.9)	− 23.7 (− 40, − 2.9)	88,348 (73,946, 104,952)	8.9 (7.3, 10.6)	3964 (3318.6, 4648.7)	− 27.9 (− 43.3, − 7.1)
Qatar	726 (532, 938)	16.4 (14.3, 18.8)	206.7 (155.5, 261.9)	− 33.4 (− 47.3, − 16.1)	24,545 (18,512, 31,356)	5.5 (4.3, 6.7)	3215.7 (2498, 4061.6)	− 43.7 (− 56.4, − 27.7)
Saudi Arabia	28,995 (22,416, 35,157)	22.6 (19.8, 25.4)	209.6 (167.6, 250.7)	− 10.1 (− 29.8, 15.4)	892,166 (683,424, 1,104,656)	10.7 (8.8, 12.6)	4430.8 (3521.4, 5282.8)	− 9.7 (− 30.3, 17.2)
Sudan	50,501 (40,471, 63,404)	25 (21, 29.5)	318 (259.9, 391.6)	− 16 (− 29.8, 3.7)	1,232,289 (952,343, 1,590,908)	9.7 (7.6, 12.6)	6465.3 (5167.5, 8161.9)	− 21.2 (− 36.4, − 0.1)
Syrian Arab Republic	25,568 (18,608, 33,483)	30.2 (26, 34.6)	265.6 (195.7, 340.2)	− 14.3 (− 36.3, 15.4)	611,300 (444,486, 807,731)	15.6 (12.7, 19)	5126.6 (3761.2, 6668.8)	− 21.9 (− 42.9, 7.9)
Tunisia	19,680 (14,437, 25,631)	29.1 (24.3, 33.6)	175.5 (128.1, 227.8)	− 16.7 (− 37.4, 10.2)	407,100 (303,333, 533,956)	14.2 (11.4, 17.1)	3312.7 (2472.4, 4317.6)	− 17.5 (− 37.8, 9.9)
Turkey	109,162 (86,561, 135,369)	24 (20.6, 27.4)	133.6 (104.8, 165.3)	− 41.2 (− 53.5, − 28.2)	2,165,550 (1,742,482, 2,645,359)	11 (9.1, 13.1)	2503.3 (2014.3, 3054.9)	− 46.4 (− 57.5, − 34)
United Arab Emirates	5983 (4354, 7968)	20.6 (16.9, 24)	215.3 (168.2, 266.2)	− 43.6 (− 55, − 30.4)	230,979 (167,478, 307,409)	10.8 (8.4, 13.4)	4415.1 (3446.5, 5592.7)	− 41.5 (− 54.3, − 26.5)
Yemen	30,564 (23,444, 39,710)	17.5 (14.5, 20.4)	280.1 (216.9, 354)	− 12.7 (− 32.2, 13.5)	777,761 (584,885, 1,025,499)	6.7 (5.3, 8.3)	5737.5 (4412.1, 7387.1)	− 15.7 (− 36.4, 12.8)

DALY: disability adjusted life year; GBD: Global Burden of Disease; ASRs: age-standardised rates.

### Individual country level

**Figure 1:**
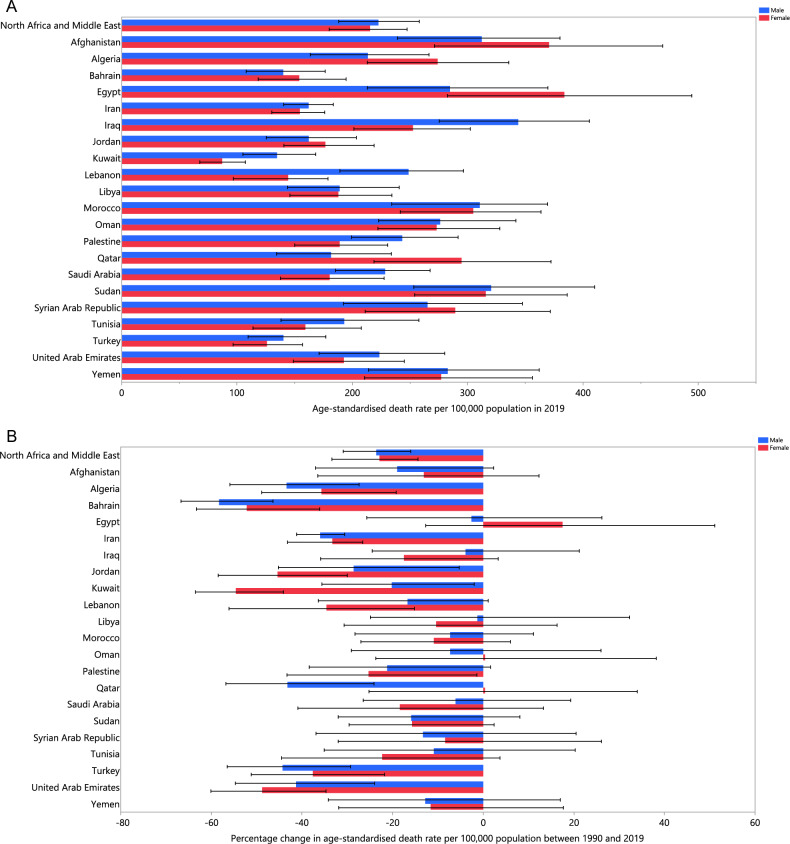
Age-standardised death rate (per 100,000 population) in 2019 (**A**) and the percentage change in the age-standardised death rate from 1990 to 2019 (**B**) of diseases and injuries attributable to high systolic blood pressure in the Middle East and North Africa region, by sex and country. (Generated from data available from http://ghdx.healthdata.org/gbd-results-tool).

The proportion of deaths attributed to HSBP in 2019 ranged from 14.3% to 35%. The three countries with the highest PAFs were Morocco (35.0% [30.6 to 39.4]), Iraq (31.0% [27.8 to 33.9] and the Syrian Arab Republic (30.2% [26.0 to 34.6]). Conversely, the MENA nations with the lowest PAFs included Afghanistan (14.3% [11.8 to 16.7]), Qatar (16.4% [14.3 to 18.8], and Yemen (17.5% [14.5 to 20.4]) (Table 1). In 2019, the age-standardised death rate due to HSBP ranged from 115.3 to 341.8 per 100,000. That same year, Afghanistan (341.8 [262.2 to 421.6]), Sudan (318.0 [259.9 to 391.6]), and Egypt (314.9 [238.4 to 403.5]) had the highest age-standardised death rates, while Kuwait (115.3 [92.9 to 138.7]), Turkey (133.6 [104.8 to 165.3]), and Bahrain (147.8 [115.6 to 184.6]) had the lowest (Table 1). Figure 1A shows the age-standardised HSBP-attributable mortality rates in 2019 by sex, which have not increased significantly in any of the MENA countries since 1990. Bahrain (− 55.5% [− 64.2 to − 43.5]), the United Arab Emirates (− 43.6% [− 55.0 to − 30.4]), and Turkey (− 41.2% [− 53.5 to − 28.8]) had the biggest declines in the age-standardised mortality rates since 1990 (Table S3). Figure 1B displays the change in the age-standardised mortality rates for males and females from 1990 to 2019.

In 2019, the proportion of DALYs that were due to HSBP varied from 5.5% to 17.9%. The three countries with the highest PAF were Morocco (17.9% [14.7 to 21.0]), Egypt (16.0% [13.1 to 18.9]), and the Syrian Arab Republic (15.6% [12.7 to 19.0]). Conversely, the smallest PAFs were found in Qatar (5.5% [4.3 to 6.7]), Afghanistan (6.0% [4.6 to 7.4]), and Yemen (6.7% [5.3 to 8.3]) (Table 1). In 2019, the age-standardised DALY rate caused by HSBP varied between 2379.7 and 7400.8 (per 100,000). The largest age-standardised DALY rates were observed in Afghanistan (7400.8 [5554.9 to 9271.6]), Egypt (6576.1 [4975.9 to 8308.1]), and Sudan (6465.3 [5167.5 to 8161.9]), with Kuwait (2379.7 [1967.1 to 2861.6]), Turkey (2503.3 [2014.3 to 3054.9]), and Bahrain (2622.3 [2110.9 to 3205]) having the lowest rates (Table 1). Figure S1 illustrates the age-standardised DALY rates in 2019 by sex. No countries recorded significant rises in the age-standardised DALY rates from 1990 to 2019. The greatest decreases were recorded in Bahrain (− 59.2% [− 67.1 to − 47.7]), Turkey (− 46.4% [− 57.5 to − 34.0]), and Qatar (− 43.7% [− 56.4 to − 27.7]) (Table 1). Figure S2 shows the percentage changes in the age-standardised DALY rates for both sexes between 1990 and 2019.

### Sex and age patterns

**Figure 2:**
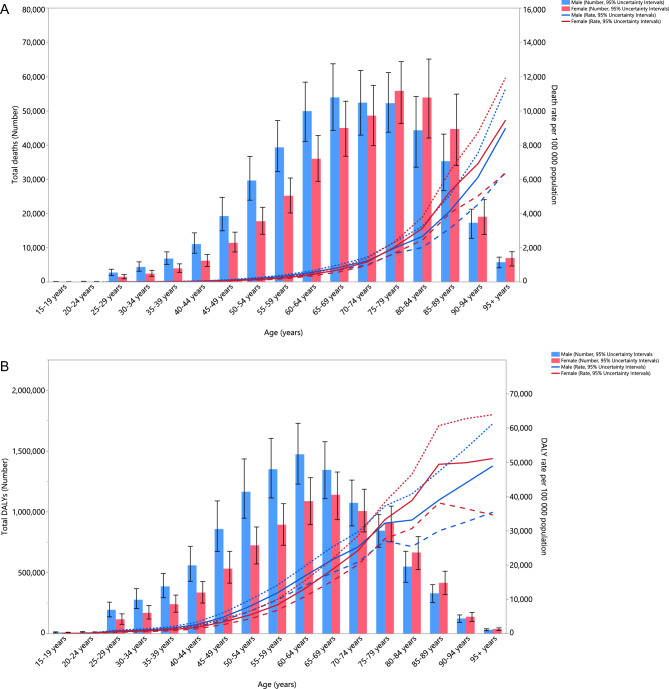
Numbers of deaths and death rate (**A**) and number of DALYs and DALY rate (**B**) of diseases and injuries attributable to high systolic blood pressure in the Middle East and North Africa region, by age and sex in 2019 Dotted and dashed lines indicate 95% upper and lower uncertainty intervals, respectively. DALY = Disability-adjusted life-year. (Generated from data available from http://ghdx.healthdata.org/gbd-results-tool).

In 2019, the HSBP-related mortality and DALY rates rose with age for both sexes. Moreover, no significant sex differences in the HSBP-related death and DALY rates were observed in any age category (Fig. 2A and B).

In 2019, the HSBP-related death cases rose with age in both sexes, with the highest number of cases identified in individuals aged 75–79 years. Moreover, males had more HSBP-related death cases than females in those aged 40–59 years old (Fig. 2A). Furthermore, for both sexes the HSBP-related DALYs increased with age up to those aged 60–64, and then declined with advancing age. In addition, HSBP-related DALYs were higher among males in those aged 50–59 years old (Fig. 2B).

### Underlying causes

**Figure 3:**
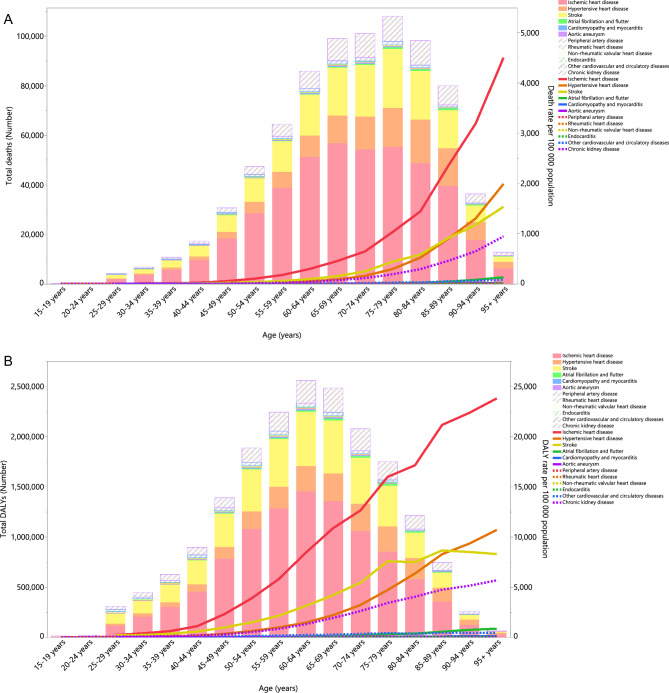
Numbers of deaths and death rate (**A**) and number of DALYs and DALY rate (**B**) of diseases and injuries attributable to high systolic blood pressure in the Middle East and North Africa region, by age and cause in 2019. DALY = Disability-adjusted life-year. (Generated from data available from http://ghdx.healthdata.org/gbd-results-tool).

In 2019, individuals aged 25 and above had the highest number of HSBP-attributable deaths and death rate from ischemic heart disease, stroke, and hypertensive heart. Notably, the number of fatalities and mortality rate due to stroke exceeded those caused by hypertensive heart disease for those aged 85–89 years old. In addition, the death rates from all HSBP-related diseases increased with age (Fig. 3A). This pattern was also found in the DALYs and the DALY rate from all diseases caused by HSBP in 2019 (Fig. 3B).

### Relationship with Socio-Demographic Index (SDI)

**Figure 4:**
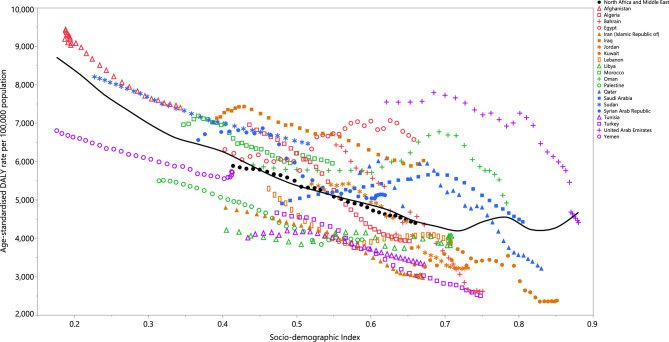
Age-standardised DALY rates of diseases and injuries attributable high systolic blood pressure for the 21 Middle East and North Africa (MENA) countries by Socio-demographic Index, 2019; the black line shows the expected values, based upon the Socio-demographic Index and disease rates. Data points above the solid black line showed a higher-than-expected burden, while those below the line showed a lower than expected burden (both based on SDI). DALY = disability-adjusted life years.

In the period 1990–2019, SDI had a negative linear relationship with the age-standardised DALY rates attributed to HSBP. Those nations that were above the solid black line exceeded the expected burden, while a lower than expected burden was exhibited for the nations underneath the line. The age-standardised DALY rates shrank for the majority of countries between 1990 and 2019. Throughout the measuring period, burdens were higher than anticipated in Afghanistan, Egypt, Morocco, Oman, Sudan, the Syrian Arab Republic, and Iraq, while burdens were lower than anticipated in Iran, Kuwait, Lebanon, Libya, Palestine, Tunisia, Turkey, and Yemen (Fig. 4).

## Discussion

This study utilized the methodological framework and data drawn from the GBD study 2019 to examine the HSBP-attributable burden in MENA from 1990 to 2019. The results shed light on the significant impact that HSBP has on the mortality and DALYs in the region. In 2019, it was estimated that HSBP was responsible for approximately 803.6 thousand deaths in the MENA region, and made up 25.9% of all deaths in MENA. Moreover, the total number of DALYs caused by HSBP in 2019 was 19.0 million, accounting for 11.6% of all DALYs. There was a decline of 23.4% in the DALYs attributable to HSBP from 1990 to 2019, indicating some progress in addressing this health issue. Our findings further emphasize the impact of HSBP on cardiovascular health in the MENA region, particularly with regards to ischemic heart disease, hypertensive heart disease, and stroke. Furthermore, an inverse relationship was identified between the age-standardised DALY rates due to HSBP and the SDI. This implies that nations with higher development levels and better access to healthcare resources tend to have a lower disease burdens from HSBP^3^.

Awareness, treatment, and control of HSBP are lower in nations that have lower SDIs than they are in nations with higher SDIs^18^. Low and middle SDI regions have increasing HSBP-related burdens, primarily because of limited awareness and fewer resources available for preventing, screening, and intervening^19,20^. Lifestyle factors can also play roles in the differences between countries in terms of the association between SDI and HSBP-attributable burden. In this regard, the burden of diseases attributable to low physical activity increased with SDI up to about high-middle SDI, while it decreased in high SDI countries^21^. Moreover, evaluating the burden of cardiovascular diseases attributable to salt intake showed that middle-SDI countries had the highest values in 2019 compared with other SDI quintiles^22^. Although there are well-known evidence-based interventions for HSBP, and its related diseases, their implementation in low to middle income countries faces several challenges at the population level^19,23,24^. These challenges include cost considerations, limited financial resources, a lack of health insurance and facilities, infrequent interactions with healthcare providers, and the unavailability of some medicines^19^. Furthermore, expanding the coverage of treatment and enhancing its community-level impact can significantly alleviate the health burden associated with HSBP^19,23^. Effectively managing HSBP requires a holistic approach, encompassing both population level awareness campaigns and individual lifestyle modifications, particularly in resource constrained regions^25^. Consistent and affordable healthcare services can improve hypertension awareness, prevention, treatment, control, and compliance. However, additional research is required to evaluate the efficacy of these methods, identify which populations and situations they are most effective in, and to assess their impact on inequality, as some people may not have access to healthcare services that are available to those with higher incomes and education.

Our findings showed that over the last three decades, the burden of diseases attributable to HSBP significantly decreased in the MENA region. It might be explained by multiple factors influence the occurrence of hypertension in this area, including high levels of smoking, obesity, sedentary habits, certain gender disparities, and an inadequate healthcare infrastructure. Additionally, socio-economic elements and discrepancies in education, literacy, and urban development exert a considerable impact^26^. To maintain or enhance the trend, other initiatives such as enhancing the accessibility and affordability of fresh fruits/vegetables, reducing the sodium levels in processed foods and common staples like bread, and enhancing the availability of salt alternatives in diets can be considered across the entire population^25^. Moreover, the adherence of patients to treatment emerges as a crucial aspect for controlling blood pressure and should be resolved^26^.

The link between hypertension and sodium intake is well-established and supported by multiple studies^27–30^. By reducing dietary sodium intake, individuals can decrease their blood pressure and risk of hypertension, in addition to reducing their chances of developing cardiovascular diseases and related health complications^27,28,31^. A systematic review and meta-analysis of 113 studies discovered that reducing dietary sodium intake led to a notable decrease in systolic blood pressure in adults^32^. In addition, reducing tobacco use, another substantial risk factor for cardiovascular diseases, is crucial for improving hypertension control and preventing cardiovascular diseases^33,34^. Therefore, a comprehensive strategy involving multiple components can be implemented to prevent, treat, and control hypertension in MENA.

Analysing the age-specific DALY rates revealed a common trend in both men and women, with rates progressively rising in younger age groups and reaching their peak among those aged 95 years and older. This suggests that HSBP has a cumulative effect over time, leading to a higher disease burden in older age groups. Men face higher exposure to social and environmental risk factors, including smoking tobacco, alcohol consumption, and unhealthy dietary habits^35^. Furthermore, they exhibit lower levels of awareness about hypertension and receive antihypertensive treatment less often than females^36^. These results underscore the need for specialised interventions and healthcare services that are tailored to the unique needs of different population subgroups.

While there have been no prior studies specifically addressing the HSBP burden in the MENA region, a number of global and country-specific studies have been published. Chen et al. used GBD 2019 to reported the all cause-specific burden that was due to HSBP^3^. They found a significant reduction in the global burden of HSBP-associated mortality from 1990 to 2019^3^. They also found that the burden linked to HSBP exhibited substantial regional, age, and sex variations^3^. Additionally, they also showed that in 2019, the HSBP-related burden was more pronounced in low and low-middle SDI regions, in comparison to regions with higher SDIs^3^. Furthermore, the age-standardised mortality rates for stroke and ischemic heart disease were also higher in areas with middle level SDIs, such as the Middle East^3^. However, they did not provide detailed estimates for the individual MENA countries.

Our findings have substantial implications for public health policy and planning in this region. They underscore the importance of early detection, prevention and management of hypertension, especially in individuals aged 25 and older, given the elevated mortality rates from ischemic heart disease associated with high systolic blood pressure. The high burden of HSBP necessitates a comprehensive and multifaceted approach to tackle this public health challenge. Efforts should promote regular blood pressure screening, lifestyle modifications, and effective pharmacological interventions to decrease blood pressure and to lower the likelihood of cardiovascular events^37–39^. Strategies should emphasize lifestyle modifications, including the promotion of smoking cessation, regularly engaging in physical exercise and maintaining a healthy diet^37–39^. Healthcare systems should be strengthened to ensure the timely diagnosis, effective treatment, and continuous management of HSBP^40^, with a particular focus on vulnerable populations and older people. Policies should address the social determinants of health, aiming to reduce inequalities and to improve access to healthcare services^41,42^. Early screening for hypertension can help identify it earlier and to reduce hypertension related health issues^43^, even though further research is required to examine the effectiveness of these strategies and to improve the accuracy of the screening methods^44,45^. This highlights the need for comprehensive cardiovascular health programs that address stroke prevention and the specific challenges associated with hypertensive heart disease in older age groups^2,46,47^.

### Strengths and limitations

This research offers valuable insights into the burden of diseases associated with HSBP in MENA, but there are also several limitations that need to be acknowledged. Firstly, the performance of the model used to estimate the data produce wide UIs for some countries, which was primarily due to the limited availability of data. Nonetheless, our main objective was to report the most accurate estimations possible by utilising data from countries with a high degree of similarity. As a result, the national results provided here should be interpreted with caution, particularly for Afghanistan, Sudan, and Yemen, due to the devastation caused by war and civil instability. Secondly, the study solely focused on the burden attributable to HSBP and did not explore other risk factors or comorbidities that may have contributed to the burden of cardiovascular diseases in MENA. Future research is required to comprehensively assess the impact of cardiovascular diseases and their related risk factors. Thirdly, the analysis was based on data from 1990 to 2019, although more recent data may provide additional insights into the changing HSBP burden in MENA. Fourthly, the GBD project only reported the burden of diseases attributable to HSBP, so we did not have the data to evaluate the burden of diseases attributable to high diastolic blood pressure. We also acknowledge the previously described methodological shortcomings of the GBD 2019 study^10,11^.

## Conclusions

The MENA region has a substantial burden of diseases attributable to HSBP. The findings underscore the need for comprehensive efforts to prevent and manage HSBP, considering its significant impact on mortality and DALYs. The observed decline in the DALY rates attributable to HSBP indicates that there has been some progress in addressing this important public health issue, although the burden remains large. Policymakers and healthcare professionals should prioritise interventions that effectively promote the early detection of HSBP, access to quality healthcare, and lifestyle modifications to reduce the burden of HSBP. Addressing the risk factors and implementing evidence-based strategies can mitigate the adverse health outcomes associated with HSBP, which will lead to improvements in the overall health and well-being of those living in the MENA region.

### Supplementary Information

Supplementary Figure S1.

Supplementary Figure S2.

Supplementary Table S1.

Supplementary Table S2.

Supplementary Table S3.

Supplementary Table S4.

## Data Availability

The data used for these analyses are all publicly available at http://vizhub.healthdata.org/gbd-results/. This study, based on publicly accessible data, exclusively expresses the authors' opinions and does not represent those of the Institute for Health Metrics and Evaluation.
