# Supplementary material for: Burden of diseases due to high systolic blood pressure in the Middle East and North Africa region from 1990 to 2019
Source: Sci Rep. 2024 Jun 13;14:13617. doi: 10.1038/s41598-024-64563-x (PMC11176357; doi:10.1038/s41598-024-64563-x)
Supplement: Supplementary file 2 — Supplementary Figure S2. [file 41598_2024_64563_MOESM2_ESM.docx]

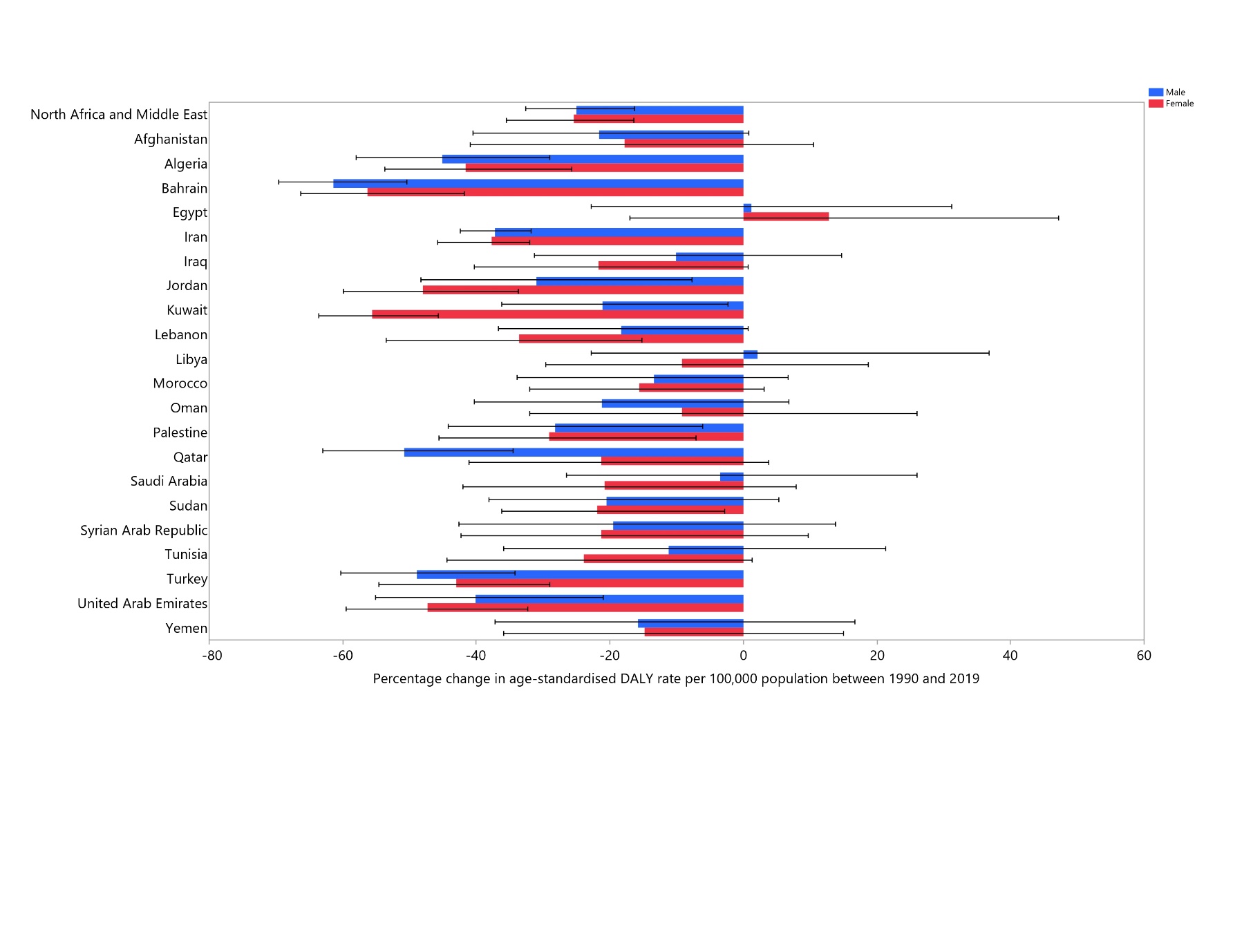


**Figure S2.** The percentage change in the age-standardised DALYs attributable to high systolic blood pressure in the Middle East and North Africa region from 1990 to 2019, by sex and country. DALY=disability-adjusted-life-year. (Generated from data available from <http://ghdx.healthdata.org/gbd-results-tool>).
