# Supplementary material for: Burden of diseases due to high systolic blood pressure in the Middle East and North Africa region from 1990 to 2019
Source: Sci Rep. 2024 Jun 13;14:13617. doi: 10.1038/s41598-024-64563-x (PMC11176357; doi:10.1038/s41598-024-64563-x)
Supplement: Supplementary file 3 — Supplementary Table S1. [file 41598_2024_64563_MOESM3_ESM.doc]

| **Table S1: Deaths attributable to high systolic blood pressure in the Middle East and North Africa region in 2019 by sex**  **(Generated from data available from http://ghdx.healthdata.org/gbd-results-tool)** | | | | | | | | |
| --- | --- | --- | --- | --- | --- | --- | --- | --- |
|  | **Male** | | | | **Female** | | | |
|  | **No**  **(95% UI)** | **PAF**  **(95% UI)** | **ASRs per 100,000 (95% UI)** | **% change in ASRs per 100,000**  **1990-2019** | **No**  **(95% UI)** | **PAF**  **(95% UI)** | **ASRs per 100,000 (95% UI)** | **% change in ASRs per 100,000**  **1990-2019** |
| **North Africa and Middle East** | **424735 (362085 , 491078)** | **24.2 (21.3 , 26.9)** | **222.7 (188.2 , 258)** | **-23.6 (-30.9 , -16)** | **378897 (320799 , 435522)** | **28.2 (24.7 , 31.2)** | **215.3 (180 , 247.5)** | **-22.9 (-33.4 , -14.4)** |
| **Afghanistan** | **16179 (12285 , 20172)** | **12.8 (10.4 , 15.1)** | **312.2 (239.1 , 380)** | **-19 (-37 , 2.3)** | **19838 (14469 , 25762)** | **15.8 (12.4 , 19.1)** | **370.5 (271.1 , 469.1)** | **-13.1 (-36.5 , 12.3)** |
| **Algeria** | **28862 (22191 , 36246)** | **27 (22.3 , 31.4)** | **213.4 (163.6 , 266.4)** | **-43.4 (-55.9 , -27.4)** | **29740 (23437 , 36316)** | **31.5 (25.9 , 36.8)** | **273.9 (213.1 , 335.6)** | **-35.7 (-48.9 , -19.2)** |
| **Bahrain** | **498 (381 , 632)** | **19 (16.4 , 22.2)** | **140.1 (107.9 , 176.5)** | **-58.3 (-66.7 , -46.4)** | **327 (254 , 412)** | **19.8 (16.3 , 23.7)** | **153.9 (118.4 , 194.6)** | **-52.2 (-63.3 , -36.1)** |
| **Egypt** | **87822 (65235 , 115674)** | **26.9 (22.2 , 31.8)** | **284.6 (213 , 369.5)** | **-2.6 (-25.7 , 26.2)** | **76888 (56675 , 98867)** | **32.7 (26.1 , 37.9)** | **383.8 (282.5 , 494.3)** | **17.5 (-12.7 , 51.1)** |
| **Iran** | **53491 (46734 , 60346)** | **23.8 (20.8 , 26.6)** | **162.1 (140.5 , 183.4)** | **-36 (-41.2 , -30.6)** | **46448 (39687 , 52480)** | **27.9 (23.7 , 31.6)** | **154.5 (130.2 , 176)** | **-33.3 (-43.2 , -26.6)** |
| **Iraq** | **31600 (24704 , 38099)** | **30.3 (27.3 , 33.2)** | **343.8 (275.3 , 405.5)** | **-3.9 (-24.5 , 21.2)** | **24116 (19180 , 29346)** | **32.1 (28.1 , 35.6)** | **252.6 (201.3 , 302.2)** | **-17.5 (-35.9 , 3.3)** |
| **Jordan** | **4435 (3409 , 5618)** | **24 (20.7 , 27.1)** | **162.1 (125.3 , 203.5)** | **-28.6 (-45.2 , -5.3)** | **3733 (2964 , 4688)** | **27.1 (23.2 , 30.6)** | **176.6 (140.6 , 218.8)** | **-45.4 (-58.5 , -30)** |
| **Kuwait** | **1859 (1463 , 2332)** | **26.3 (22.6 , 29.8)** | **134.7 (105.1 , 168.2)** | **-20.2 (-35.6 , -2)** | **675 (528 , 826)** | **22.9 (19 , 26.4)** | **87.1 (67.7 , 107.3)** | **-54.6 (-63.5 , -44.1)** |
| **Lebanon** | **5689 (4330 , 6781)** | **30.2 (23.4 , 34.9)** | **248.7 (189.2 , 296.4)** | **-16.7 (-36.4 , 1.1)** | **4068 (2713 , 5044)** | **27.1 (18.1 , 33)** | **144.3 (96.8 , 178.9)** | **-34.6 (-56.1 , -15.2)** |
| **Libya** | **4456 (3417 , 5751)** | **24.8 (21.2 , 29.6)** | **189 (143.9 , 240.6)** | **-1.3 (-24.9 , 32.3)** | **4198 (3256 , 5208)** | **30.6 (26 , 34.9)** | **187.9 (145.8 , 234.3)** | **-10.4 (-30.7 , 16.3)** |
| **Morocco** | **40030 (29947 , 48067)** | **32.3 (27.3 , 37.1)** | **310.4 (234.1 , 369.3)** | **-7.3 (-28.3 , 11.1)** | **39874 (31640 , 47570)** | **38.3 (32.7 , 43.1)** | **304.8 (241.5 , 363.5)** | **-10.9 (-27 , 6)** |
| **Oman** | **1682 (1376 , 2058)** | **21.4 (18.2 , 24.8)** | **276.1 (222.7 , 341.7)** | **-7.3 (-29.1 , 26)** | **1313 (1083 , 1562)** | **29.2 (24.5 , 33.6)** | **273.1 (222.2 , 327.9)** | **0.4 (-23.7 , 38.2)** |
| **Palestine** | **2000 (1652 , 2404)** | **22.4 (19.4 , 25.2)** | **243.2 (199.3 , 291.6)** | **-21.2 (-38.4 , 1.6)** | **1816 (1470 , 2212)** | **23.6 (19.5 , 27.8)** | **189 (150 , 230.6)** | **-25.3 (-43.3 , -1.4)** |
| **Qatar** | **536 (386 , 707)** | **16.1 (13.8 , 18.6)** | **181.5 (134.3 , 233.9)** | **-43.2 (-56.8 , -24.1)** | **190 (144 , 245)** | **17.5 (14.3 , 20.6)** | **294.7 (218.7 , 372.2)** | **0.4 (-25.2 , 34)** |
| **Saudi Arabia** | **19453 (15144 , 23716)** | **22.8 (19.7 , 25.8)** | **228.5 (185.4 , 267.4)** | **-6.1 (-26.5 , 19.3)** | **9542 (7218 , 12055)** | **22.3 (18.6 , 25.5)** | **180.3 (137.7 , 227.5)** | **-18.4 (-40.9 , 13.3)** |
| **Sudan** | **27978 (21738 , 36242)** | **23.9 (19.6 , 28.8)** | **320.3 (253.2 , 410)** | **-15.9 (-32 , 8.1)** | **22523 (18065 , 28067)** | **26.6 (22 , 31.5)** | **315.7 (254 , 386.3)** | **-15.7 (-29.6 , 2.4)** |
| **Syrian Arab Republic** | **14313 (10298 , 19321)** | **29.5 (24.9 , 34)** | **265.2 (192.3 , 347.4)** | **-13.3 (-36.9 , 20.5)** | **11256 (8278 , 14794)** | **31.3 (25.8 , 36.8)** | **289.2 (211.1 , 371.6)** | **-8.4 (-32 , 26.1)** |
| **Tunisia** | **10292 (7291 , 13985)** | **26.9 (21.9 , 31.5)** | **193 (138.1 , 257.7)** | **-10.9 (-35.1 , 20.3)** | **9388 (6797 , 12228)** | **32 (25.5 , 37.8)** | **159.2 (113.9 , 207.8)** | **-22.3 (-44.5 , 3.7)** |
| **Turkey** | **52692 (40978 , 66590)** | **21.3 (18.4 , 24.4)** | **140.3 (109.6 , 177)** | **-44.3 (-56.5 , -29.3)** | **56470 (43215 , 70255)** | **27.1 (22.5 , 31.4)** | **126 (96.6 , 156.8)** | **-37.6 (-51.2 , -21.8)** |
| **United Arab Emirates** | **4994 (3552 , 6751)** | **20.8 (16.6 , 24.6)** | **223.4 (171.2 , 280.1)** | **-41.3 (-54.7 , -24)** | **988 (738 , 1295)** | **19.6 (16.5 , 22.7)** | **192.6 (148.9 , 245)** | **-48.8 (-60.1 , -34.7)** |
| **Yemen** | **15443 (11594 , 20358)** | **15.7 (12.5 , 18.7)** | **282.7 (214 , 362)** | **-12.8 (-34.2 , 17)** | **15121 (11432 , 19645)** | **19.8 (16.1 , 23.4)** | **277 (210.5 , 356.1)** | **-11.6 (-31.9 , 17.7)** |
