# Supplementary material for: Burden of diseases due to high systolic blood pressure in the Middle East and North Africa region from 1990 to 2019
Source: Sci Rep. 2024 Jun 13;14:13617. doi: 10.1038/s41598-024-64563-x (PMC11176357; doi:10.1038/s41598-024-64563-x)
Supplement: Supplementary file 4 — Supplementary Table S2. [file 41598_2024_64563_MOESM4_ESM.doc]

| **Table S2: DALYs attributable to high systolic blood pressure in the Middle East and North Africa region in 2019 by sex**  **(Generated from data available from http://ghdx.healthdata.org/gbd-results-tool)** | | | | | | | | |
| --- | --- | --- | --- | --- | --- | --- | --- | --- |
|  | **Male** | | | | **Female** | | | |
|  | **No**  **(95% UI)** | **PAF**  **(95% UI)** | **ASRs per 100,000 (95% UI)** | **% change in ASRs per 100,000**  **1990-2019** | **No**  **(95% UI)** | **PAF**  **(95% UI)** | **ASRs per 100,000 (95% UI)** | **% change in ASRs per 100,000**  **1990-2019** |
| **North Africa and Middle East** | **10589945 (8982944 , 12302416)** | **12.1 (10.5 , 13.7)** | **4670.2 (3994.3 , 5387.8)** | **-25 (-32.6 , -16.3)** | **8438088 (7194474 , 9702506)** | **11.2 (9.5 , 12.9)** | **4105.8 (3505.6 , 4687)** | **-25.4 (-35.5 , -16.4)** |
| **Afghanistan** | **467690 (352440 , 611980)** | **5.4 (4.1 , 6.7)** | **6848.7 (5220.4 , 8450.4)** | **-21.6 (-40.5 , 0.8)** | **552076 (390212 , 745485)** | **6.6 (4.8 , 8.5)** | **7923.1 (5830.2 , 10253.1)** | **-17.8 (-40.9 , 10.5)** |
| **Algeria** | **631049 (483816 , 790872)** | **12.4 (9.9 , 14.9)** | **3833.8 (2956.3 , 4759.2)** | **-45.1 (-58 , -29)** | **591900 (473189 , 724669)** | **11.8 (9.5 , 14.2)** | **4143.3 (3307.1 , 4990.5)** | **-41.6 (-53.7 , -25.7)** |
| **Bahrain** | **15977 (12543 , 19784)** | **9.3 (7.4 , 11.2)** | **2578.1 (2050.1 , 3187.8)** | **-61.4 (-69.6 , -50.4)** | **8285 (6619 , 10272)** | **7.4 (5.7 , 9.1)** | **2617.8 (2076.5 , 3251.9)** | **-56.3 (-66.3 , -41.8)** |
| **Egypt** | **2331830 (1732834 , 3083520)** | **16.1 (13.2 , 19.4)** | **6418.9 (4800.2 , 8349.7)** | **1.2 (-22.8 , 31.2)** | **1894833 (1385429 , 2434570)** | **15.9 (12.4 , 19.1)** | **7070.1 (5305 , 8977.5)** | **12.8 (-17 , 47.2)** |
| **Iran** | **1218371 (1091596 , 1348440)** | **11.5 (9.9 , 13.2)** | **3295.7 (2949.8 , 3650.7)** | **-37.2 (-42.4 , -31.8)** | **908895 (806424 , 1012537)** | **9.9 (8.2 , 11.6)** | **2657.4 (2331.5 , 2961.1)** | **-37.7 (-45.8 , -32)** |
| **Iraq** | **811676 (630438 , 1012380)** | **14.5 (12.2 , 16.8)** | **7100.9 (5609.2 , 8599)** | **-10.1 (-31.3 , 14.7)** | **569782 (450724 , 705575)** | **12.1 (9.9 , 14.5)** | **4982.4 (4016 , 6048.5)** | **-21.7 (-40.3 , 0.7)** |
| **Jordan** | **116960 (90585 , 146836)** | **10.3 (8.3 , 12.3)** | **3342.9 (2614.1 , 4157.7)** | **-31 (-48.3 , -7.7)** | **84199 (67828 , 105220)** | **8.4 (6.8 , 10)** | **3090.3 (2490.1 , 3814.3)** | **-48 (-59.9 , -33.7)** |
| **Kuwait** | **52528 (41155 , 65679)** | **12.3 (10 , 14.7)** | **2918.1 (2325.8 , 3627.4)** | **-21.1 (-36.2 , -2.3)** | **16647 (13487 , 20314)** | **5.1 (4 , 6.3)** | **1628.4 (1321.5 , 1975.7)** | **-55.6 (-63.6 , -45.7)** |
| **Lebanon** | **125535 (97858 , 148240)** | **18.2 (14.2 , 21.1)** | **5300.6 (4138.5 , 6257.7)** | **-18.3 (-36.7 , 0.7)** | **78240 (54499 , 95118)** | **11.9 (8.2 , 14.6)** | **2738.5 (1905.7 , 3324.4)** | **-33.6 (-53.5 , -15.2)** |
| **Libya** | **116273 (90337 , 153883)** | **12.8 (10.5 , 16.1)** | **4142.4 (3208.9 , 5313.7)** | **2.1 (-22.8 , 36.8)** | **101672 (80039 , 124683)** | **12.8 (10.4 , 15.2)** | **3969.4 (3138.6 , 4885.5)** | **-9.2 (-29.6 , 18.7)** |
| **Morocco** | **919698 (682813 , 1124583)** | **17.9 (14.6 , 21.4)** | **6094 (4587.8 , 7340.8)** | **-13.4 (-33.9 , 6.7)** | **883994 (697121 , 1078608)** | **17.8 (14.7 , 21.1)** | **5811.4 (4629.5 , 6963)** | **-15.6 (-32 , 3.1)** |
| **Oman** | **49105 (40179 , 60190)** | **9 (7.3 , 11.3)** | **4928.5 (4069.4 , 5944.3)** | **-21.2 (-40.3 , 6.8)** | **31446 (26232 , 37031)** | **10.1 (8.1 , 12.3)** | **4890.9 (4065.8 , 5783.2)** | **-9.2 (-32 , 26)** |
| **Palestine** | **50618 (42072 , 61135)** | **9.7 (7.9 , 11.5)** | **4593.3 (3837.8 , 5483.2)** | **-28.2 (-44.2 , -6.1)** | **37730 (31169 , 45267)** | **8 (6.4 , 9.8)** | **3387.2 (2781.7 , 4091.3)** | **-29.1 (-45.6 , -7.1)** |
| **Qatar** | **19297 (14451 , 24831)** | **5.9 (4.6 , 7.3)** | **2908.6 (2223.5 , 3708.3)** | **-50.8 (-63 , -34.5)** | **5248 (3999 , 6714)** | **4.4 (3.3 , 5.6)** | **4174.3 (3188.4 , 5187.2)** | **-21.3 (-41.1 , 3.8)** |
| **Saudi Arabia** | **606233 (461879 , 759178)** | **11.8 (9.6 , 13.9)** | **4894.9 (3947.5 , 5810.8)** | **-3.5 (-26.5 , 26)** | **285932 (216603 , 362517)** | **9 (7 , 11.2)** | **3709.3 (2890.4 , 4615.7)** | **-20.8 (-42 , 7.9)** |
| **Sudan** | **696888 (521154 , 923950)** | **10.2 (7.7 , 13.5)** | **6714.3 (5154.8 , 8793.9)** | **-20.5 (-38.1 , 5.3)** | **535401 (420613 , 683885)** | **9.2 (7.1 , 11.8)** | **6208.9 (5047.1 , 7663.5)** | **-21.9 (-36.2 , -2.8)** |
| **Syrian Arab Republic** | **355937 (257964 , 482504)** | **17.2 (13.9 , 20.7)** | **5606 (4101.8 , 7524.9)** | **-19.5 (-42.6 , 13.8)** | **255363 (186305 , 339460)** | **13.8 (10.8 , 17.2)** | **4831.1 (3618 , 6191.4)** | **-21.3 (-42.3 , 9.7)** |
| **Tunisia** | **224533 (161918 , 302498)** | **14.8 (11.8 , 17.8)** | **3743.5 (2709.4 , 5011.9)** | **-11.2 (-35.9 , 21.3)** | **182566 (136992 , 236639)** | **13.5 (10.4 , 16.5)** | **2899.6 (2164.5 , 3759.8)** | **-23.9 (-44.4 , 1.3)** |
| **Turkey** | **1168277 (916333 , 1456875)** | **11.5 (9.6 , 13.5)** | **2826.6 (2214.4 , 3520.9)** | **-48.9 (-60.3 , -34.2)** | **997273 (798686 , 1216023)** | **10.5 (8.4 , 12.7)** | **2174.9 (1742.7 , 2650.1)** | **-43 (-54.6 , -29)** |
| **United Arab Emirates** | **195014 (138608 , 263422)** | **11.8 (9.1 , 14.8)** | **4662.8 (3574.3 , 5941.2)** | **-40.1 (-55.1 , -21)** | **35965 (26615 , 47180)** | **7.3 (5.5 , 9.1)** | **3664.2 (2900.4 , 4618.3)** | **-47.3 (-59.5 , -32.3)** |
| **Yemen** | **405695 (295818 , 548029)** | **6.6 (5.1 , 8.3)** | **5932.1 (4443.8 , 7780.2)** | **-15.8 (-37.2 , 16.7)** | **372066 (279630 , 492481)** | **6.8 (5.3 , 8.6)** | **5543.3 (4235.8 , 7174.1)** | **-14.8 (-35.9 , 15)** |
