# Supplementary material for: Burden of diseases due to high systolic blood pressure in the Middle East and North Africa region from 1990 to 2019
Source: Sci Rep. 2024 Jun 13;14:13617. doi: 10.1038/s41598-024-64563-x (PMC11176357; doi:10.1038/s41598-024-64563-x)
Supplement: Supplementary file 5 — Supplementary Table S3. [file 41598_2024_64563_MOESM5_ESM.doc]

| **Table S3: Deaths attributable to high systolic blood pressure in the Middle East and North Africa region in 1990 and 2019**  **(Generated from data available from http://ghdx.healthdata.org/gbd-results-tool)** | | | | | | | |
| --- | --- | --- | --- | --- | --- | --- | --- |
|  | **1990** | | | **2019** | | | **% change in ASRs per 100,000**  **1990-2019** |
|  | **No**  **(95% UI)** | **PAF**  **(95% UI)** | **ASRs per 100,000 (95% UI)** | **No**  **(95% UI)** | **PAF**  **(95% UI)** | **ASRs per 100,000 (95% UI)** |
| **North Africa and Middle East** | **408873 (361884 , 458664)** | **16.1 (14.3 , 17.8)** | **286.5 (246.7 , 323.4)** | **803631 (687099 , 923807)** | **25.9 (22.9 , 28.6)** | **219.4 (185.6 , 252.5)** | **-23.4 (-31.5 , -15.9)** |
| **Afghanistan** | **25633 (19987 , 31667)** | **14.1 (11.7 , 16.5)** | **405 (319.9 , 490.8)** | **36017 (27027 , 45488)** | **14.3 (11.8 , 16.7)** | **341.8 (262.2 , 421.6)** | **-15.6 (-35 , 5.1)** |
| **Algeria** | **32361 (26203 , 38912)** | **21 (18 , 24)** | **393 (315.8 , 472.6)** | **58602 (46555 , 71314)** | **29.1 (24.7 , 33.3)** | **235.5 (185.6 , 287)** | **-40.1 (-51.8 , -26)** |
| **Bahrain** | **425 (352 , 503)** | **21.6 (18.6 , 24.6)** | **332.4 (273.2 , 394.6)** | **825 (652 , 1031)** | **19.3 (16.8 , 22.4)** | **147.8 (115.6 , 184.6)** | **-55.5 (-64.2 , -43.5)** |
| **Egypt** | **73780 (62665 , 84327)** | **15.9 (13.4 , 18.3)** | **309.7 (262.3 , 358.7)** | **164710 (123672 , 209933)** | **29.3 (24.4 , 33.7)** | **314.9 (238.4 , 403.5)** | **1.7 (-22 , 28.4)** |
| **Iran (Islamic Republic of)** | **47648 (41787 , 53977)** | **13.4 (11.6 , 15.3)** | **244.8 (209.5 , 279.6)** | **99939 (86758 , 112458)** | **25.6 (22.1 , 28.8)** | **157.8 (135.3 , 179.1)** | **-35.6 (-42.3 , -31.3)** |
| **Iraq** | **23216 (19374 , 27603)** | **19.5 (17 , 22.1)** | **332 (275.1 , 396.1)** | **55716 (44030 , 67478)** | **31 (27.8 , 33.9)** | **296.6 (238.4 , 350)** | **-10.7 (-29 , 8.8)** |
| **Jordan** | **2796 (2343 , 3275)** | **18.7 (16.2 , 21.1)** | **276.4 (231.7 , 323.6)** | **8168 (6704 , 9751)** | **25.3 (22.3 , 28)** | **168.5 (137.8 , 199.5)** | **-39 (-50.5 , -26.5)** |
| **Kuwait** | **896 (782 , 1004)** | **16.8 (14.6 , 18.9)** | **182.8 (156.2 , 206.3)** | **2534 (2062 , 3083)** | **25.3 (21.8 , 28.4)** | **115.3 (92.9 , 138.7)** | **-36.9 (-46.9 , -25.7)** |
| **Lebanon** | **4997 (4037 , 6085)** | **22.2 (18.9 , 25.7)** | **259.4 (209.2 , 317.5)** | **9757 (7074 , 11640)** | **28.8 (21.2 , 33.8)** | **191.3 (138.6 , 228.4)** | **-26.3 (-45.9 , -12)** |
| **Libya** | **3343 (2689 , 4088)** | **16.9 (14.4 , 19.5)** | **200.5 (160.4 , 243.3)** | **8654 (6726 , 10717)** | **27.3 (23.5 , 31.3)** | **188.7 (146.1 , 232.3)** | **-5.9 (-26.1 , 20.9)** |
| **Morocco** | **39040 (33552 , 44449)** | **22 (19.2 , 24.8)** | **339.1 (290.1 , 384.2)** | **79904 (61938 , 93834)** | **35 (30.6 , 39.4)** | **307.7 (240 , 359.3)** | **-9.3 (-25.7 , 5)** |
| **Oman** | **1494 (1118 , 1955)** | **15.2 (12.5 , 18.3)** | **287.5 (215.2 , 371.2)** | **2996 (2540 , 3494)** | **24.2 (21 , 27.3)** | **274.5 (225.1 , 326.8)** | **-4.5 (-24.1 , 23.6)** |
| **Palestine** | **2110 (1644 , 2607)** | **17.2 (14.6 , 20)** | **277.4 (216.8 , 343.4)** | **3816 (3182 , 4529)** | **23 (19.9 , 26)** | **211.6 (174.5 , 252.9)** | **-23.7 (-40 , -2.9)** |
| **Qatar** | **213 (174 , 259)** | **16.2 (13.9 , 18.7)** | **310.2 (245.2 , 372.2)** | **726 (532 , 938)** | **16.4 (14.3 , 18.8)** | **206.7 (155.5 , 261.9)** | **-33.4 (-47.3 , -16.1)** |
| **Saudi Arabia** | **11447 (9013 , 14378)** | **13.8 (11.5 , 16.2)** | **233.2 (184 , 287.9)** | **28995 (22416 , 35157)** | **22.6 (19.8 , 25.4)** | **209.6 (167.6 , 250.7)** | **-10.1 (-29.8 , 15.4)** |
| **Sudan** | **30834 (25127 , 37339)** | **12.1 (10.2 , 14.3)** | **378.3 (308.7 , 454.7)** | **50501 (40471 , 63404)** | **25 (21 , 29.5)** | **318 (259.9 , 391.6)** | **-16 (-29.8 , 3.7)** |
| **Syrian Arab Republic** | **14344 (11152 , 17566)** | **19.5 (16.3 , 22.7)** | **310 (239.4 , 379.5)** | **25568 (18608 , 33483)** | **30.2 (26 , 34.6)** | **265.6 (195.7 , 340.2)** | **-14.3 (-36.3 , 15.4)** |
| **Tunisia** | **8485 (7027 , 10101)** | **19.7 (16.6 , 22.8)** | **210.7 (173.4 , 251.8)** | **19680 (14437 , 25631)** | **29.1 (24.3 , 33.6)** | **175.5 (128.1 , 227.8)** | **-16.7 (-37.4 , 10.2)** |
| **Turkey** | **71532 (60490 , 82380)** | **18.3 (15.7 , 20.8)** | **227.2 (189.4 , 261.9)** | **109162 (86561 , 135369)** | **24 (20.6 , 27.4)** | **133.6 (104.8 , 165.3)** | **-41.2 (-53.5 , -28.2)** |
| **United Arab Emirates** | **1116 (902 , 1399)** | **19.4 (16.6 , 23.3)** | **381.6 (317.1 , 461.8)** | **5983 (4354 , 7968)** | **20.6 (16.9 , 24)** | **215.3 (168.2 , 266.2)** | **-43.6 (-55 , -30.4)** |
| **Yemen** | **12888 (9866 , 16551)** | **8.7 (7.1 , 10.5)** | **320.8 (250.1 , 401.6)** | **30564 (23444 , 39710)** | **17.5 (14.5 , 20.4)** | **280.1 (216.9 , 354)** | **-12.7 (-32.2 , 13.5)** |
