# Supplementary material for: Burden of diseases due to high systolic blood pressure in the Middle East and North Africa region from 1990 to 2019
Source: Sci Rep. 2024 Jun 13;14:13617. doi: 10.1038/s41598-024-64563-x (PMC11176357; doi:10.1038/s41598-024-64563-x)
Supplement: Supplementary file 6 — Supplementary Table S4. [file 41598_2024_64563_MOESM6_ESM.doc]

| **Table S4: DALYs attributable to high systolic blood pressure in the Middle East and North Africa region in 1990 and 2019**  **(Generated from data available from http://ghdx.healthdata.org/gbd-results-tool)** | | | | | | | |
| --- | --- | --- | --- | --- | --- | --- | --- |
|  | **1990** | | | **2019** | | | **% change in ASRs per 100,000**  **1990-2019** |
|  | **No**  **(95% UI)** | **PAF**  **(95% UI)** | **ASRs per 100,000 (95% UI)** | **No**  **(95% UI)** | **PAF**  **(95% UI)** | **ASRs per 100,000 (95% UI)** |
| **North Africa and Middle East** | **10107979 (8966338 , 11314423)** | **6.1 (5.4 , 6.8)** | **5887.9 (5221.2 , 6582.3)** | **19028033 (16327380 , 21927273)** | **11.6 (10.1 , 13.3)** | **4401.9 (3785.9 , 5042.8)** | **-25.2 (-33.9 , -16.8)** |
| **Afghanistan** | **663575 (505362 , 836465)** | **5.7 (4.5 , 6.9)** | **9186.5 (7100.1 , 11432.1)** | **1019766 (760082 , 1334770)** | **6 (4.6 , 7.4)** | **7400.8 (5554.9 , 9271.6)** | **-19.4 (-39.4 , 4.5)** |
| **Algeria** | **776023 (625856 , 937601)** | **8 (6.7 , 9.4)** | **6958.7 (5663.2 , 8314.9)** | **1222949 (966991 , 1502570)** | **12.1 (10 , 14.4)** | **3927.6 (3132.5 , 4790)** | **-43.6 (-55.7 , -28.9)** |
| **Bahrain** | **11917 (9929 , 14153)** | **9.6 (8 , 11.4)** | **6431.6 (5370.2 , 7560.6)** | **24262 (19467 , 29874)** | **8.5 (6.9 , 10.3)** | **2622.3 (2110.9 , 3205)** | **-59.2 (-67.1 , -47.7)** |
| **Egypt** | **1851844 (1576131 , 2117565)** | **6.5 (5.5 , 7.6)** | **6314.8 (5392.8 , 7203.6)** | **4226663 (3191278 , 5446888)** | **16 (13.1 , 18.9)** | **6576.1 (4975.9 , 8308.1)** | **4.1 (-21.1 , 32.3)** |
| **Iran (Islamic Republic of)** | **1205799 (1054681 , 1364300)** | **4.8 (4.1 , 5.6)** | **4800.2 (4200.5 , 5423.5)** | **2127266 (1913281 , 2347736)** | **10.8 (9.1 , 12.4)** | **2973.4 (2652.2 , 3280.2)** | **-38.1 (-43.8 , -33.8)** |
| **Iraq** | **562681 (468925 , 669831)** | **7.2 (6.1 , 8.3)** | **7130.7 (5973.4 , 8436.6)** | **1381459 (1078019 , 1706965)** | **13.4 (11.2 , 15.6)** | **6025.4 (4796.3 , 7308.9)** | **-15.5 (-33.8 , 5.2)** |
| **Jordan** | **69399 (58690 , 81425)** | **6.8 (5.7 , 7.8)** | **5417.3 (4592.3 , 6301.4)** | **201159 (168057 , 239001)** | **9.4 (7.9 , 11)** | **3228.8 (2707.6 , 3804.5)** | **-40.4 (-51.7 , -27.1)** |
| **Kuwait** | **25393 (22056 , 28805)** | **6.3 (5.4 , 7.3)** | **3748.2 (3277.5 , 4207.5)** | **69176 (56649 , 83667)** | **9.2 (7.5 , 11.1)** | **2379.7 (1967.1 , 2861.6)** | **-36.5 (-46.1 , -25.1)** |
| **Lebanon** | **118891 (98173 , 144392)** | **9.9 (8.3 , 11.8)** | **5295.5 (4364.9 , 6438.3)** | **203776 (153914 , 240750)** | **15.1 (11.3 , 17.7)** | **3904.4 (2956.7 , 4605.3)** | **-26.3 (-44.2 , -9.6)** |
| **Libya** | **80673 (65345 , 98682)** | **6 (5.1 , 7.1)** | **4212.3 (3440.2 , 5106.9)** | **217945 (172545 , 273901)** | **12.8 (10.6 , 15.3)** | **4062 (3223.6 , 5040.7)** | **-3.6 (-24.8 , 23.7)** |
| **Morocco** | **949470 (820826 , 1091475)** | **8.5 (7.3 , 9.7)** | **6962 (6017.3 , 7928.4)** | **1803693 (1394123 , 2176429)** | **17.9 (14.7 , 21)** | **5955.2 (4659.1 , 7044.1)** | **-14.5 (-30.7 , 1.5)** |
| **Oman** | **39979 (29554 , 52415)** | **6.4 (5.1 , 8.1)** | **5912.2 (4456.8 , 7656.7)** | **80551 (68395 , 94411)** | **9.4 (7.8 , 11.3)** | **4921.4 (4161.2 , 5715.3)** | **-16.8 (-34.3 , 10.2)** |
| **Palestine** | **46685 (36117 , 58076)** | **6.1 (4.9 , 7.4)** | **5499.9 (4290.1 , 6812.8)** | **88348 (73946 , 104952)** | **8.9 (7.3 , 10.6)** | **3964 (3318.6 , 4648.7)** | **-27.9 (-43.3 , -7.1)** |
| **Qatar** | **6454 (5153 , 7976)** | **6.3 (5 , 7.6)** | **5707.9 (4698.8 , 6810.1)** | **24545 (18512 , 31356)** | **5.5 (4.3 , 6.7)** | **3215.7 (2498 , 4061.6)** | **-43.7 (-56.4 , -27.7)** |
| **Saudi Arabia** | **300199 (230582 , 383714)** | **5.6 (4.4 , 6.9)** | **4907.2 (3876.3 , 6131.4)** | **892166 (683424 , 1104656)** | **10.7 (8.8 , 12.6)** | **4430.8 (3521.4 , 5282.8)** | **-9.7 (-30.3 , 17.2)** |
| **Sudan** | **785992 (636889 , 966261)** | **4.3 (3.5 , 5.2)** | **8206.8 (6668.9 , 9926.5)** | **1232289 (952343 , 1590908)** | **9.7 (7.6 , 12.6)** | **6465.3 (5167.5 , 8161.9)** | **-21.2 (-36.4 , -0.1)** |
| **Syrian Arab Republic** | **361191 (285143 , 446713)** | **7.6 (6.1 , 9.1)** | **6561.1 (5182.5 , 8043.4)** | **611300 (444486 , 807731)** | **15.6 (12.7 , 19)** | **5126.6 (3761.2 , 6668.8)** | **-21.9 (-42.9 , 7.9)** |
| **Tunisia** | **195304 (160973 , 233381)** | **7.3 (6 , 8.6)** | **4013.3 (3323 , 4753.2)** | **407100 (303333 , 533956)** | **14.2 (11.4 , 17.1)** | **3312.7 (2472.4 , 4317.6)** | **-17.5 (-37.8 , 9.9)** |
| **Turkey** | **1671852 (1421002 , 1931523)** | **7.1 (6.1 , 8.2)** | **4667 (3957.1 , 5361)** | **2165550 (1742482 , 2645359)** | **11 (9.1 , 13.1)** | **2503.3 (2014.3 , 3054.9)** | **-46.4 (-57.5 , -34)** |
| **United Arab Emirates** | **37692 (30084 , 48043)** | **8.7 (7.1 , 10.8)** | **7550.9 (6332.9 , 9072.4)** | **230979 (167478 , 307409)** | **10.8 (8.4 , 13.4)** | **4415.1 (3446.5 , 5592.7)** | **-41.5 (-54.3 , -26.5)** |
| **Yemen** | **340166 (256709 , 445178)** | **3 (2.4 , 3.8)** | **6804.9 (5235.5 , 8710.6)** | **777761 (584885 , 1025499)** | **6.7 (5.3 , 8.3)** | **5737.5 (4412.1 , 7387.1)** | **-15.7 (-36.4 , 12.8)** |
